# Novel Decellularization Method for Tissue Slices

**DOI:** 10.3389/fbioe.2022.832178

**Published:** 2022-03-09

**Authors:** Maria Narciso, Anna Ulldemolins, Constança Júnior, Jorge Otero, Daniel Navajas, Ramon Farré, Núria Gavara, Isaac Almendros

**Affiliations:** ^1^ Unitat de Biofísica i Bioenginyeria, Facultat de Medicina i Ciències de la Salut, Universitat de Barcelona, Barcelona, Spain; ^2^ The Institute for Bioengineering of Catalonia (IBEC), The Barcelona Institute of Science and Technology, Barcelona, Spain; ^3^ CIBER de Enfermedades Respiratorias, Madrid, Spain; ^4^ Institut d’Investigacions Biomèdiques August Pi i Sunyer, Barcelona, Spain

**Keywords:** decellularization, bioscaffold recellularization, biocompatibility, extracellular matrix, tissue slices, lung

## Abstract

Decellularization procedures have been developed and optimized for the entire organ or tissue blocks, by either perfusion of decellularizing agents through the tissue’s vasculature or submerging large sections in decellularizing solutions. However, some research aims require the analysis of native as well as decellularized tissue slices side by side, but an optimal protocol has not yet been established to address this need. Thus, the main goal of this work was to develop a fast and efficient decellularization method for tissue slices—with an emphasis on lung—while attached to a glass slide. To this end, different decellularizing agents were compared for their effectiveness in cellular removal while preserving the extracellular matrix. The intensity of DNA staining was taken as an indicator of remaining cells and compared to untreated sections. The presence of collagen, elastin and laminin were quantified using immunostaining and signal quantification. Scaffolds resulting from the optimized protocol were mechanically characterized using atomic force microscopy. Lung scaffolds were recellularized with mesenchymal stromal cells to assess their biocompatibility. Some decellularization agents (CHAPS, triton, and ammonia hydroxide) did not achieve sufficient cell removal. Sodium dodecyl sulfate (SDS) was effective in cell removal (1% remaining DNA signal), but its sharp reduction of elastin signal (only 6% remained) plus lower attachment ratio (32%) singled out sodium deoxycholate (SD) as the optimal treatment for this application (6.5% remaining DNA signal), due to its higher elastin retention (34%) and higher attachment ratio (60%). Laminin and collagen were fully preserved in all treatments. The SD decellularization protocol was also successful for porcine and murine (mice and rat) lungs as well as for other tissues such as the heart, kidney, and bladder. No significant mechanical differences were found before and after sample decellularization. The resulting acellular lung scaffolds were shown to be biocompatible (98% cell survival after 72 h of culture). This novel method to decellularize tissue slices opens up new methodological possibilities to better understand the role of the extracellular matrix in the context of several diseases as well as tissue engineering research and can be easily adapted for scarce samples like clinical biopsies.

## 1 Introduction

The extracellular matrix (ECM) plays an important role by regulating cell behavior through structural and biochemical stimulation. The ECM is composed of more than 300 core structural components (Burgstaller et al., 2017) that provide physical and chemical cues that regulate essential cellular mechanisms (Gattazzo et al., 2014), including proliferation, migration and differentiation. Overall, the ECM is divided into two major compartments: the interstitial ECM and the basement membrane. The interstitial ECM is in the intercellular spaces and is composed mostly of fibrous proteins and polysaccharides, most predominantly collagen type I and III, elastin, and fibronectin. The basement membrane is made up of sheets of deposition of ECM components—mainly collagen type IV and laminins—that are located under epithelial and endothelial cells (Pompili et al., 2021). These two layers make up the “core matrisome.” However, the ECM has other components such as ECM-affiliated proteins and secreted factors that are typically removed during decellularization and thus less frequently characterized (Hynes and Naba 2012). The study of the ECM and its characteristics yields an important understanding of the complex interactions between cells and their microenvironment, which plays a pivotal role in various diseases including cancer and fibrosis (Rendin et al., 2019; Wishart et al., 2020; Júnior et al., 2021). Accordingly, decellularized ECM scaffolds have great potential for tissue engineering and regenerative medicine. In fact, decellularized tissues can be used for the generation of ECM hydrogels (Falcones et al., 2021), for the recellularization of whole acellular organs (Ohata and Ott 2020), as well as applications in tissue regeneration (Zhu et al., 2019). Thus, it is unsurprising the growing interest to work on physiomimetic tissue scaffolds by decellularizing different types of tissues (Mendibil et al., 2020).

To remove cells from their ECM, several strategies can be employed (Mendibil et al., 2020) by using physical, chemical, enzymatic or a combination of approaches. Physical strategies include freeze/thawing cycles, which induce crystals in the matrix disrupting the cell membrane, as well as agitation, which is commonly used in conjunction with another/s decellularizing agent/s facilitating cell lysis. Chemical strategies include detergents, such as Sodium Dodecyl Sulfate (SDS) or Sodium Deoxycholate (SD), that solubilize the cell membrane and hypertonic or hypotonic solutions, that will cause cell disruption by osmotic shock. Finally, enzymatic approaches can target the adhesion of cells to the ECM, such as the combination of trypsin with EDTA (ethylenediaminetetraacetic acid) or target the nuclear material of the cell, such as with deoxyribonuclease (DNAse). In this context, each decellularizing agent can affect differently each specific ECM components. In fact, to assess the quality of the resulting scaffold it is required to analyze the effects of the decellularizing agent on the ECM components. At times, only components of the interstitial ECM are analyzed, like collagen type I and elastin (O’Neill et al., 2013a; Balestrini et al., 2015; Alshaikh et al., 2020). However, the evaluation of the basement membrane is of paramount importance especially for tissue engineering and cell culture applications since these components heavily influence cell adhesion and differentiation (Rasmussen and Karsdal 2016). The most analyzed basement membrane components include collagen type IV and laminins (Maghsoudlou et al., 2013; Zhang et al., 2016).

The available decellularization protocols have important limitations. Most of them require prolonged periods, taking from 6–7 h (Rosmark et al., 2018) to several days (Wishart et al., 2020; Wüthrich et al., 2020). Additionally, most of the available methods fail in the preservation of some ECM components. For instance, significant decreases in elastin (Maghsoudlou et al., 2013; Alshaikh et al., 2020), collagen (Hill et al., 2015; Alshaikh et al., 2020), glycosaminoglycans (Mendoza-Novelo et al., 2011; Lü et al., 2014), laminins (Hill et al., 2015; Wüthrich et al., 2020) and proteoglycans (Hill et al., 2015; Calle et al., 2016) have been described. In addition to these limitations, the current protocols are still not particularly flexible or accessible. Most decellularization procedures are suited for decellularizing a whole organ or thick sections of an organ (or blocks). However, this type of protocol would not be useful for many experimental conditions as is the case for small clinical biopsies. Indeed, this type of sample is scarce and cannot be decellularized by accessing the tissue’s vasculature. Furthermore, the full biopsy cannot often be decellularized since it is also required for later histopathological testing. Other experimental setups might require consecutive native and acellular sections of the same individual. This is the case for studies on cancer samples where the tumor location and inner structures must be identified prior to decellularization. For early-stage tumors especially, this is of paramount importance as the cancer cells are removed during decellularization and its location could not be pinpointed. As no current methods have explored the decellularization of glass-attached thin tissue sections (under 100 µm), a novel protocol that allows for the study of the exact location before and after decellularization is needed to fill this gap.

In this study, we set out to develop a novel decellularization method where a thin tissue section is kept attached to a glass slide allowing for “*in situ*ˮ decellularization without removing the sample from a microscopic stage. This method is significantly faster and less wasteful than other available methods while maintaining the mechanical properties of the sample and being suited for cell culture applications. Our findings show that this method is highly versatile and applicable to tissues of different animal origins and from different organs, such as the bladder, heart, and kidney. Additionally, it provides the option to study the same tissue section before and after decellularization, which is invaluable for studies of certain pathologies and of scarce or valuable clinical samples.

## 2 Materials and Methods

### 2.1 Organ Procurement and Sample Preparation

This work was approved by the Institutional Committee of Universitat de Barcelona and the Animal Experimentation Committee of regional authorities (Generalitat de Catalunya, OB 168/19 and 10972). The lungs were obtained from male C57BL/6J mice (10 weeks old; Charles River Laboratories, Saint Germain sur L’arbresle, France) and male Sprague-Dawley rats (body weight ∼ 300 g Charles River Laboratories, Saint Germain sur L’arbresle, France). Bladders, hearts, and kidneys were harvested from the adult mice and embedded in Optimum Cutting Temperature compound, OCT (Tissue-Tek, Sakura, Torrance, CA, United States) and immediately stored at −80°C. Porcine lungs were obtained from the local butcher and a small portion was embedded in OCT. Mice lung samples were sectioned at 20 and 100 µm using a cryostat, with a working temperature of −24°C. All other samples were sectioned only at 20 µm thickness. Slices were deposited onto a positively charged glass slide (Superfrost Plus; Thermo Fischer Scientific) and air-dried for 15 min before being stored at −80°C until further use. Before decellularization, 20 µm thick sections were thawed are room temperature for 20 min and 100 µm thick sections were thawed for 40 min. It is important to thaw the sample completely for increased attachment. The freeze-thaw cycle already functions as an initial step of decellularization (Gilpin and Yang 2017).

### 2.2 Decellularization Protocol and Methods Comparison

**FIGURE 1:**
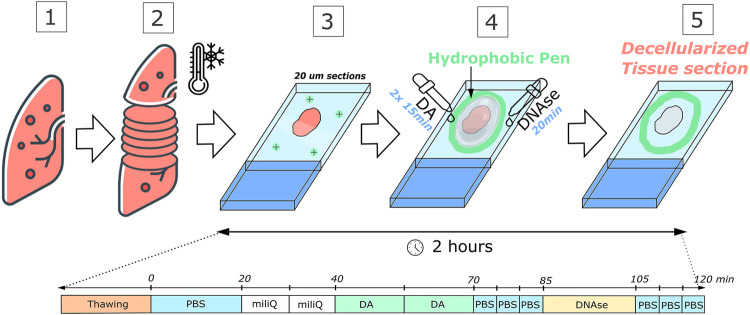
Schematic of the novel decellularization method for a lung sample. (1) Organs are harvested and stored at −80°C and embedded in OCT; (2) then they are cryosectioned into 20 µm sections and (3) deposited onto positively charged glass slides; (4) Decellularization is performed through a series of washes and rinses, including the decellularizing agent (DA) and a Deoxyribonuclease I (DNAse) incubation. After carefully removing the reagents with PBS, a fully decellularized section is produced in 2 h (5). MilliQ is a form of ultrapurified water.

Before decellularization, the area surrounding each sample was traced with a liquid repellent slide marker pen (or hydrophobic pen) to better control and reduce the volume of reagents needed (Figure 1). Acellular sections were produced by consecutive washes and rinses of the sliced section with different solutions while still firmly attached to the glass slide.

All decellularizing protocols studied in this work followed the same schedule. For 20 µm sections, an initial 20-min PBS wash to remove the OCT from the sample. To cause an osmotic shock and disrupt the cellular membrane, the next step was to apply two consecutive 10-min washes with ultrapure water. Subsequently, the decellularizing agent was incubated twice for 15 min intervals to dissolve the cellular membrane and detach the cells from the matrix. The following step was to remove the decellularizing agent with three 5-min washes of PBS. A 20-min incubation with DNAse I solution (0.3 mg/ml, 5 mM MgCl_2_, 5 mM CaCl_2_ in 1 mM Tris-HCl) was carried out to remove DNA fragments. Finally, 5-min washes (x3) of PBS were performed to remove the DNAse I solution. For 100 µm sections, DNAse I solution was incubated for 40 min at 37°C, for optimal enzymatic activity.

Six decellularizing agents (DAs) were tested and compared. The DAs and their concentrations were adapted from existing decellularization protocols, and were as follows:

*Method 1:* Ammonium Hydroxide 0.5% + Triton 0.1%, based on (Ng et al., 2019);
*Method 2:* CHAPS 0.5%, based on (Rosmark et al., 2018);
*Method 3:* Sodium Deoxycholate (SD) 2%, based on (Xiong et al., 2015);
*Method 4:* Sodium Dodecyl Sulfate (SDS) 1%, based on (Jorba et al., 2019);
*Method 5:* Triton X-100 1%, based on (Mendoza-Novelo et al., 2011);
*Method 6:* Trypsin 0.05% + EDTA 0.02%, based on (Schenke-Layland et al., 2003);

Detailed information about the existing decellularization protocols can be found in Supplementary Table S1.

### 2.3 Attachment to the Glass Slide

For this decellularization method, one key factor is the attachment of the sample to the glass slide throughout the decellularization protocol. Thus, we calculated the percentage of sample attachment (number of samples that remained attached after the treatment/total number of samples tested) × 100%. It is a visual observation as the sample is clearly detached and removed from the glass slide during decellularization. Five decellularization experiments were conducted and samples were counted before and after decellularization. For each experiment, 5, 6 sections were used per treatment.

### 2.4 Immunofluorescence, DNA and Histological Staining

Both cellular and acellular mice lung sections from Methods 1–5 were stained for laminin, type I collagen, elastin, and DNA to determine the effects of the decellularization protocol on the matrix proteins as well as the cellular material and DNA. These proteins were specifically chosen because they are three major components of the ECM: collagen and elastin can provide information on the interstitial ECM and laminin on the basement membrane. For native sections where the decellularization protocol was not followed, immunofluorescent staining was performed after consecutive washes of PBS to remove the OCT. For decellularized sections, the staining protocol was performed immediately after the decellularization procedure described above. The tissue was fixed using 4% paraformaldehyde (PFA) for 10 min at room temperature (RT). Samples were then blocked using a buffer composed of 10% fetal bovine serum (FBS) and supplemented with 3% bovine serum albumin (BSA) for 1 h at RT. Primary antibodies against elastin (rabbit anti elastin, BioNova, 1:100), type I collagen (rabbit anti-collagen type I, Abcam, 1:100), and laminin (rabbit anti laminin, Thermo Fisher Scientific, Waltham, MA, 1:100) were incubated in the same formulation of the blocking buffer overnight at 4°C and constant agitation (80 rpm). Sections were then rinsed three times with the blocking buffer. The secondary antibody (goat anti-rabbit Cy3, Thermo Fischer, 1:200) was incubated at a 1:200 dilution in the blocking buffer for 2 h, at 37°C and constant agitation (80 rpm). Three 15 min rinses with PBS were performed to eliminate the unbound secondary antibodies. DNA of cellular and acellular samples was stained by incubation with Hoechst 33342 (Thermo Fisher Scientific)—for 20 min at 80 rpm in an orbital shaker followed by three 5-min PBS washes to remove excess staining with the same agitation settings. The Hoechst staining concentration was carried out following the manufacturer’s instructions. Finally, samples were mounted in Fluoromount mounting media (Thermo Fisher Scientific) and stored at 4°C. For each experiment, laminin, type I collagen, and elastin staining was performed in consecutive lung sections. The color of the laminin and elastin fluorescent images was changed after image acquisition from red to yellow and green respectively, for easier figure readability. Brightness and contrast were improved for the same purpose. Both cellular and acellular mice lung sections from Methods 1–5 were stained using Picro-Sirius Red Stain Kit (ScyTek Laboratories, US) for collagen type I and III presence and Hematoxylin and Eosin (PanReac Applichem) to assess the presence of the nuclei.

### 2.5 Mechanical Testing by Atomic Force Microscopy

To assess mechanical changes in the tissue before and after decellularization, an Atomic Force Microscope (AFM) was used to measure the stiffness, viscosity and force adhesion of the samples. In a custom-built AFM system, the cantilever was displaced in 3D with nanometer resolution employing piezo actuators coupled to strain gauge sensors (Physik Instrumente, Germany) to measure the vertical displacement of the cantilever. The deflection of the cantilever was measured with a quadrant photodiode (S4349, Hamamatsu, Japan). The cantilevers employed had a nominal spring constant value of 0.03 N/m and a silicon oxide bead with a 4.5 µm diameter attached to its end (Novascan Technologies, IA).

The lung ECM was probed while submersed in PBS at RT. Three lung sections from different mice were measured using AFM before and after decellularization using the SD-based protocol. Before the measurements, a small region in the lung parenchyma was selected and marked with a pen. With the visual assistance of the optical microscope, the tip was positioned macroscopically over the region of interest of the lung sample. Up to 20 randomly selected locations were indented within the delineated region before decellularization and the same was performed after decellularization within the same region. Only alveolar structures were considered by excluding airways, blood vessels and the pleural region.

The deflection and displacement of the cantilever were recorded as the cantilever descended and contacted the sample surface at constant speed up to a maximum loading force, with a ramp amplitude of 15 µm and frequency of 1 Hz. To calculate the model’s parameters, each curve was fitted through a custom MATLAB code (MATLAB, The MathWorks Inc. MA, United States). The Young’s modulus fitting was performed using the approaching curve and fitting the appropriate tip-sample contact model to the force-indentation curve (Jorba et al., 2017). Viscosity measurements were computed by following the model described in (Rebelo et al., 2013) and force adhesion was obtained by computing the minimum of the retracting curves. All mechanical values were obtained by computing the mean values of the five curves recorded consecutively at each point.

### 2.6 Cell Culture

Primary human Bone Marrow-Derived Mesenchymal Stromal Cells (hBM-MSCs) (PCS-500-012, ATCC) were cultured in Mesenchymal Stromal Cell Basal Medium (PCS-500-030, ATCC) following manufacturer’s instructions at 37°C in air with 5% CO_2_ and 95% relative humidity. Cells from passages 3–6 were used for experiments.

### 2.7 Biocompatibility Assay

After decellularization, the 20 µm-thick and 100 µm-thick scaffolds were incubated for 2 h with a peracetic acid solution (269336, Sigma-Aldrich) 0, 1% (v/v) in 4% ethanol for sterilization. Subsequently, they were washed with PBS (11593377, Gibco) and 5·10^4^ cells/cm^2^ hBM-MSCs were seeded on top of the lung scaffolds. Control cultures were seeded on conventional culture plastic flasks and maintained in parallel with the same conditions. After 24, 48, and 72 h of recellularization, samples were stained using the LIVE/DEAD Viability/Cytotoxicity kit (L-3224, Invitrogen). Calcein-AM was used to indicate live cells (green), and ethidium homodimer-1 was used to indicate dead cells (red) as previously described (Bonenfant et al., 2013; O’Neill et al., 2013b; Syed et al., 2014). After 72 h, F-Actin was stained in the 100 µm scaffolds (phalloidin, Thermo Scientific, Waltham, MA, United States) and visualized by a confocal microscope. The results were obtained by counting the number of live cells from five independent biocompatibility assays.

### 2.8 Imaging and Decellularization Quantification

Epifluorescent images of the tissue sections were acquired with a Leica SP5 inverted microscope equipped with a CCD camera (C9100, Hamamatsu Photonics K.K. Hamamatsu, Japan) and using a ×10 and ×20 Plan Fluor objective (Nikon). For 3D images of the 100 µm scaffolds and cellular distribution, a Nikon D-Eclipse Ci confocal microscope was used in conjunction with a ×20 Plan Apo immersion oil objective (Nikon). For the imaging of the cytoskeleton of the cells ×20, ×60, and ×100 immersion oil objectives were used (Nikon). For the histological stains, a ×20 and a ×40 objective was used with an Olympus BX41TF upright microscope.

Decellularization quantification was performed as previously described (Narciso et al., 2021). Briefly, images belonging to a given treatment (decellularized slices) and corresponding control condition (native slices belonging to the same organ and animal) were acquired in a single imaging session. Exposure times for the phase contrast (PC) and the fluorescent channels were set based on the control (native) sections corresponding to each experiment. At least ten locations per condition were imaged except when less than ten locations were needed to cover the complete tissue area. Images were taken 1 mm apart from each other starting from the edge of a sample until covering the entirety of the sample length. The DNA signal intensity of the images from decellularized sections was compared to the DNA signal intensity of the corresponding native sections. The same method was used to quantify collagen, elastin, and laminin signals resulting from immunofluorescent staining. The signal corresponding to the ECM proteins of the untreated native sections was normalized to 100%.

### 2.9 Versatility of the Method for Different Species and Organs

To assess whether the SD decellularization method can be used for other types of tissues, 20 µm-thick sections of mice bladder, heart and kidney were subjected to the same decellularization protocol previously described in Section 2.2. Three independent decellularization experiments of each organ were performed. Additionally, three independent decellularization experiments were conducted using lung slices from pigs and rats, since these species are commonly used in animal models of respiratory diseases.

### 2.10 Statistical Analysis

For experiments with two groups (native and decellularized), statistical comparisons were performed by an unpaired two-tailed *t*-test. Unless mentioned, all data are mean ± SD. Differences were considered statistically significant for *p* < 0.05. Statistical analysis was performed using GraphPad Prism (GraphPad software 9.1.0, Inc. San Diego, CA, United States).

## 3 Results

### 3.1 SD and SDS are the Most Effective Treatments for Cellular Removal

Even though trypsin + EDTA has been previously used in decellularization protocols, this method detached all tested samples and thus it is not suited for decellularization protocols on a glass slide. For that reason, no results on DNA staining and ECM proteins are presented here for trypsin decellularization, since no samples could be stained. All other decellularization methods resulted in scaffolds suited for further staining.

**FIGURE 2:**
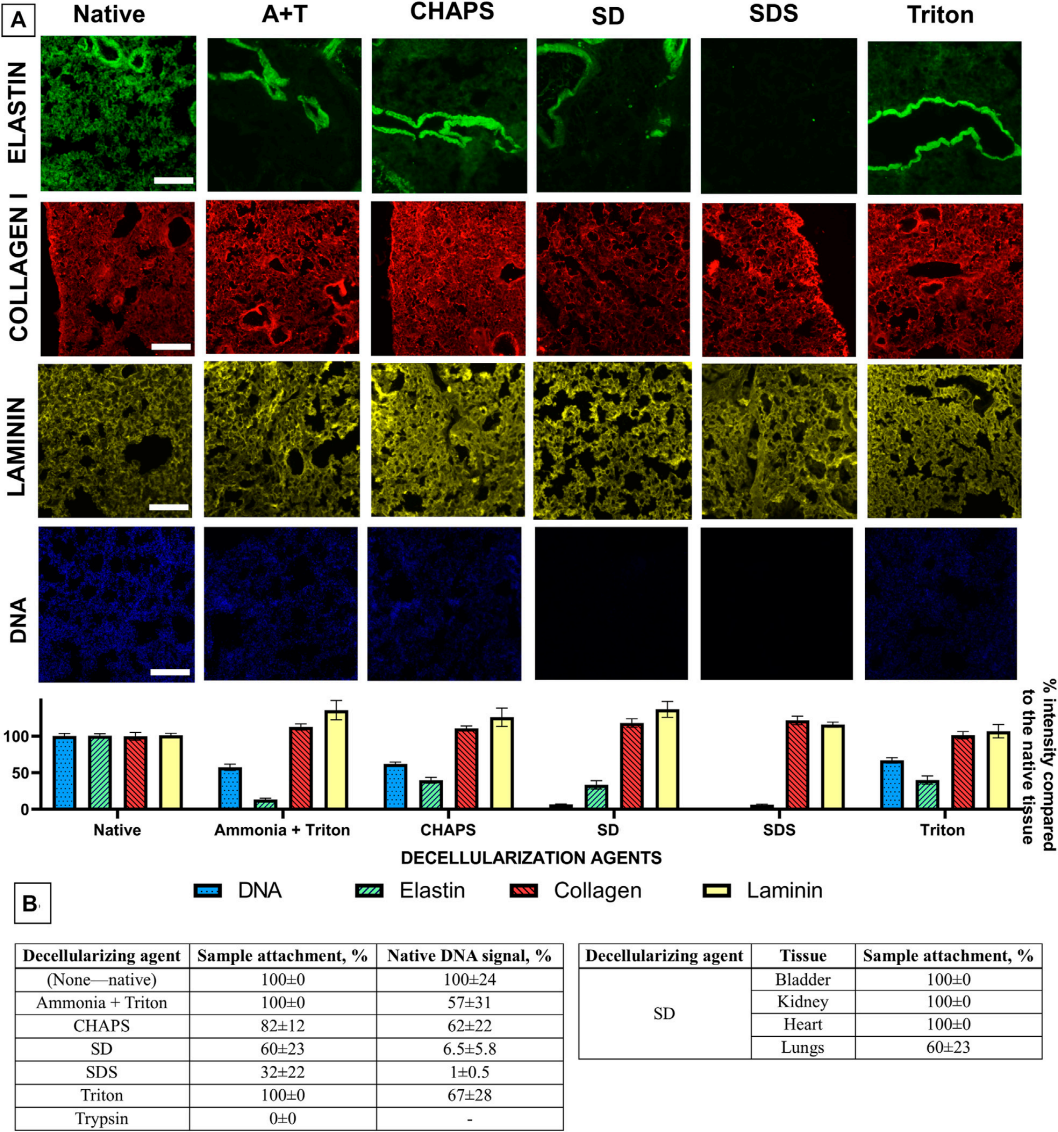
**(A)** Staining and signal quantification of 20 µm lung sections from mice for nuclei, elastin, type I collagen, and laminin. Lung sections had been previously subjected to five different decellularization treatments (Ammonia + Triton, CHAPS, SD, SDS, and Triton). Laminin and elastin fluorescent images were changed from the original red color to yellow and green respectively for easier image readability. Native tissue's signal was normalized to 100% Scale bar = 200 µm. **(B)** Table describing the attachment ratio of tissue slices from the glass slide for each decellularization treatment (left) and Table describing the attachment ratio of different tissues treated with SD (right). Data are mean ± SEM.

In Figure 2, representative UV images of Hoechst staining clearly show the removal of cell nuclei and DNA from all different methods studied. Cell nuclei are clearly present in native sections and noticeably absent (or reduced) after each decellularization treatment. To quantify the different decellularization levels resulting from different treatments, pixels corresponding to tissue were separated from background pixels, quantified, and compared to native tissue pixel intensities as previously described (Narciso et al., 2021). The DNA signal from native sections was normalized to 100% for easier comparison. All methods resulted in a significant reduction in DNA staining (*p* < 0.05) when compared to untreated native sections, as evidenced in Figure 2. SD and SDS treatments resulted in a marked DNA signal decrease (6.5 ± 5.8% and 1 ± 0.5% of native DNA signal, respectively) indicating that most cells and residual DNA was removed. On the other hand, CHAPS, Triton, and Ammonia/Triton mixture showed 62 ± 22%, 67 ± 28%, and 57 ± 31% of the native DNA signal, respectively, not reaching an acceptable decellularization level. A qualitative analysis of nuclei of lung sections stained with the hematoxylin and eosin (Supplementary Figure S1) validates the results obtained from the UV images of Hoechst33342-stained nuclei: only SDS and SD seemed to have efficiently removed cell nuclei from the sample, while the other methods did not reach sufficient cell removal rates.

### 3.2 All Treatments Preserved the ECM Proteins

As shown in Figure 2, all treatments resulted in a reduction of the elastin signal. SDS and the Ammonia and triton mixture in particular, showed a marked degradation of elastin signal where 6 ± 3% and 13 ± 8% of the native elastin signal remained, respectively. SD, CHAPS, and Triton, on the other hand, showed higher rates of elastin preservation, accounting for 34 ± 25%, 40 ± 19%, and 40 ± 24% of the pre-treatment elastin signal. This decrease is especially visible in the lung parenchyma, while blood vessels and airways seem to maintain elastin signal levels (Figure 2). Regarding laminin and collagen, none of the treatments used showed a decrease in laminin or collagen signal when compared to the corresponding native section. A quantitative analysis of samples stained with Picro-Sirius red (Supplementary Figure S2) showed a clear reduction in collagen type I and III stain in the SDS treated samples (81 ± 3%) when compared to the native samples (100% signal). Samples treated with the other decellularizing agents showed no marked differences when compared visually to the native sections: Ammonia + Triton, CHAPS, SD and Triton treatments showed 100 ± 4%, 108 ± 4%, 98 ± 3%, and 108 ± 3% when compared to the native image, respectively.

### 3.3 Samples Remained Attached to the Glass Slide

As observed in Figure 2B, treatments with higher rates of cellular removal were more likely to detach the sample from the glass slide; Ammonia + Triton, CHAPS and Triton treatments did not affect the attachment of the sample to the glass slide, for the most part, achieving 100 ± 00%, 82 ± 12%, and 100 ± 00% of sample attachment, respectively. For treatments that achieved higher cell removal—SDS and SD -, 32 ± 22% and 60 ± 23% of samples remained attached after decellularization treatments, respectively. By comparing all types of tissues, 60% of the lung sections remained attached while bladder, heart, and kidney sections never detached, achieving a sample attachment ratio of 100 ± 00% after using SD as the decellularizing agent. Taking into account the effectiveness in the removal of cellular material described in 3.1, the preservation of collagen, elastin, and laminin, and previous accounts of SDS’s effects on the matrix, SD was considered to be the best treatment for on-slide decellularization. Thus, biomechanical, biocompatibility, and versatility analysis were performed on scaffolds resulting from the SD decellularization method.

### 3.4 SD Treatment did not Significantly Change the ECM Stiffness, Viscosity or Adhesion

**FIGURE 3:**
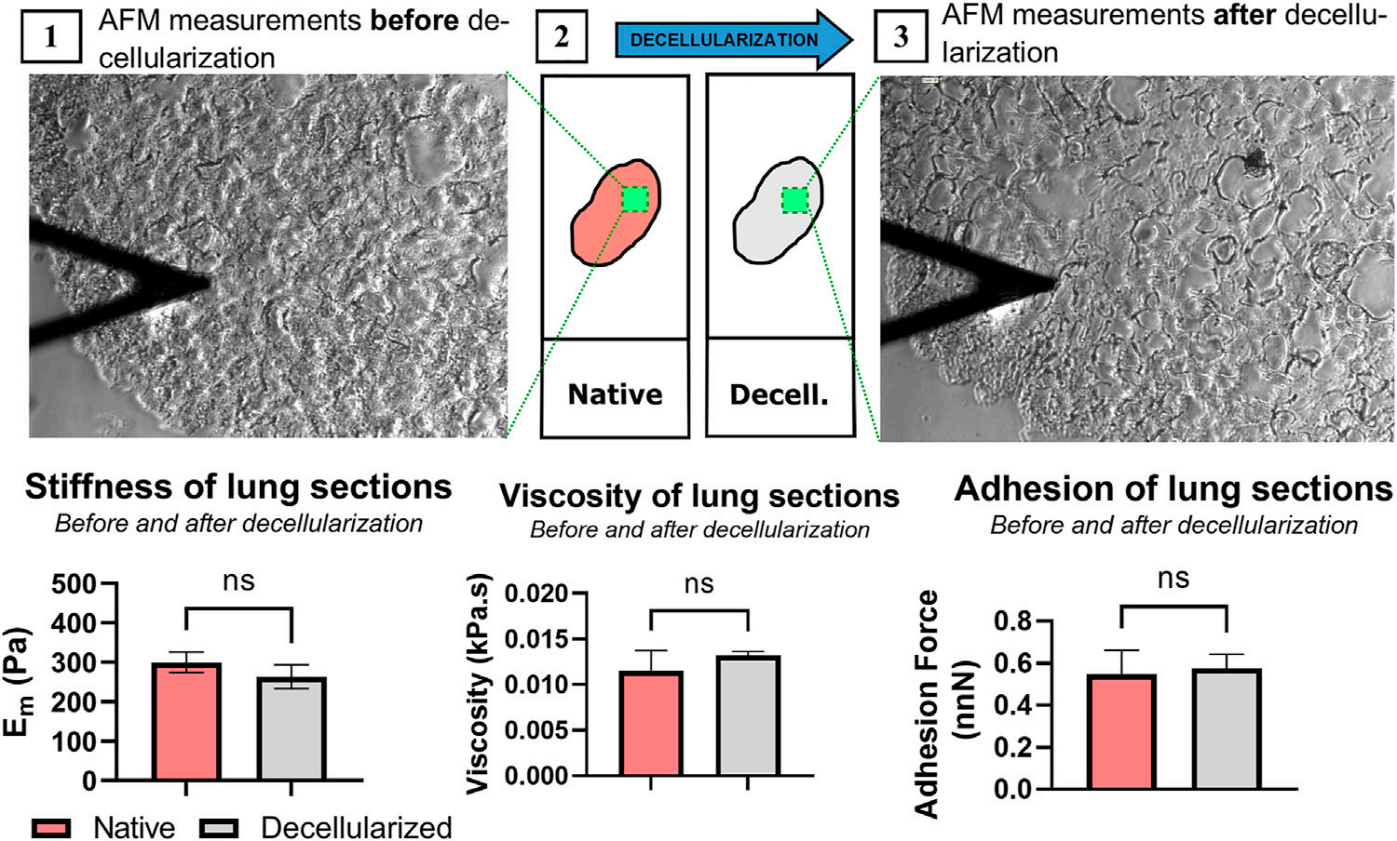
Effect of the SD decellularization protocol on the mechanical properties of the lung scaffold. **(A)** Representative image of AFM measurements of the same tissue slice before (1) and after (3) decellularization (*n* = 3). Results of stiffness, viscosity, and adhesion force of the same sample before and after decellularization. Significance was *p* < 0.05.

The mean Young’s modulus, viscosity, and force adhesion from each sample were computed from the same indentations and are shown in Figure 3. As expected, there was a slight, but not significant, decrease in the sample stiffness after decellularization. E_m_ for the native untreated samples was 299.6 ± 26.1 Pa while the E_m_ for the same sections but after decellularization was of 263.0 ± 29.9 Pa. Viscosity and force adhesion measurements were similar before and after decellularization (0.012 ± 0.002 vs. 0.013 ± 0.001 kPa·s and 0.48 ± 0.13 vs. 0.51 ± 0.01 nN, respectively). These results indicate that the decellularization protocol does not significantly change the mechanical properties of the lung sections.

### 3.5 SD Derived ECM Scaffolds are Biocompatible

**FIGURE 4:**
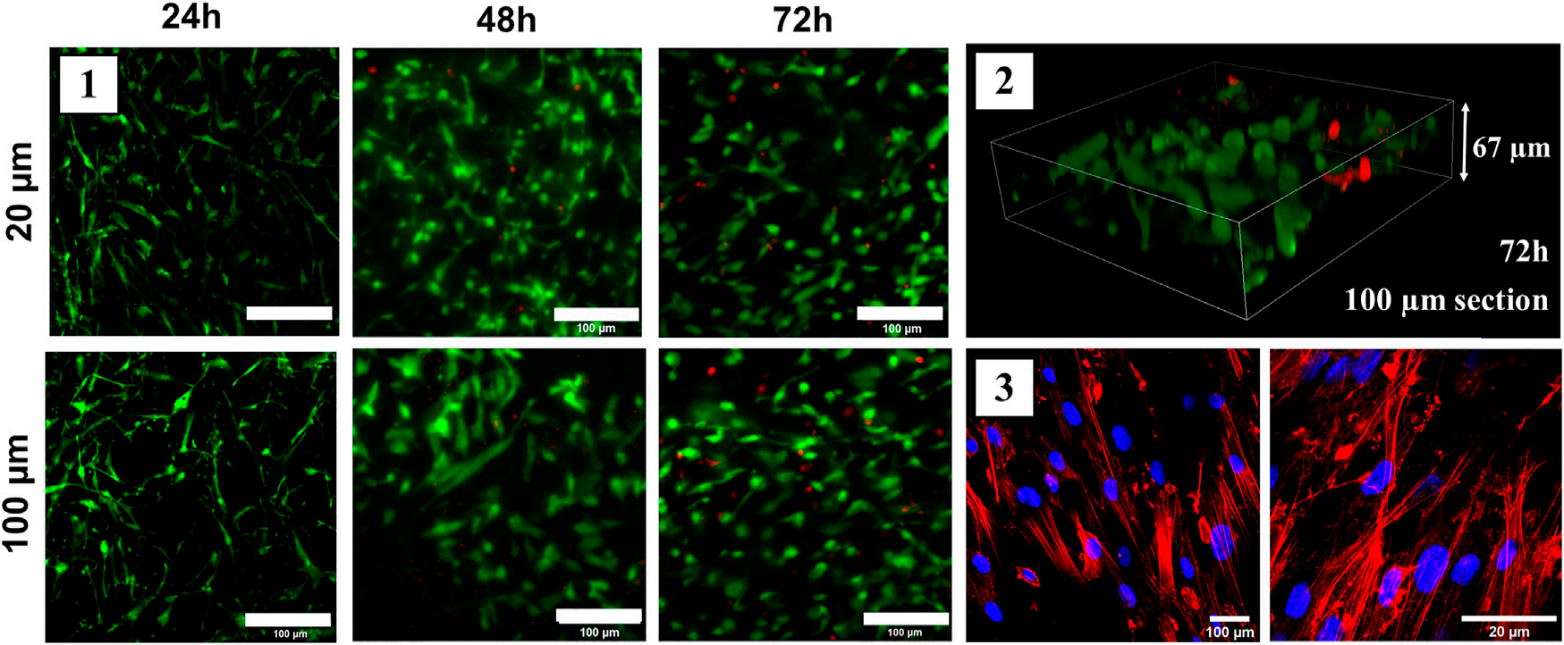
Biocompatibility of the lung scaffold. Representative images of biocompatibility assay of 20 and 100 µm scaffolds for 24, 48, and 72 h. Live cells are seen in green while dead cells are red (1); 3D image of a 100 µm scaffold seeded with MSCs after 72 h. Stack showed covers 67 µm of the scaffold (2); Phalloidin staining of actin fibers (red) of cells seeded in 100 µm lung scaffold after 72 h (3). Samples decellularized with SD.

The results of the biocompatibility assay were quantified and are detailed in Figure 4.1. Overall, cells survived similarly on both the 20 and 100 µm ECM scaffold thickness for 24, 48, and 72 h. More specifically, the viable cell ratio did not significantly differ after 24 h: at 72 h the cell viability ratio was 98.5 ± 2.0% and 97.9 ± 0.8% for 20 and 100 μm, respectively. After 72 h, the cell viability was similar to the cell count on the conventional culture flask (99.1 ± 0.8%). Accordingly, the distribution of the cells through the scaffold was assessed with a three-dimensional live/dead image and Z-distribution of the cells within the decellularized ECM structure can be seen in Figure 4.2. Alive cells were attached to the different parts of the matrix at different heights, which is an indicator of cell mobility and distribution within the lung scaffold. Previous topographical data indicates that lung scaffolds have gradual changes in height at the cellular level (around more or less 10 µm of variation), which is far below the variation in cell distribution seen in Figure 4. The cytoskeleton of the cells seeded on the matrix (Figure 4.3) shows a spread morphology indicative of their healthy attachment to the new scaffold.

### 3.6 The SD Method is Successful in Decellularizing Tissues From Different Animals and Organs

**FIGURE 5:**
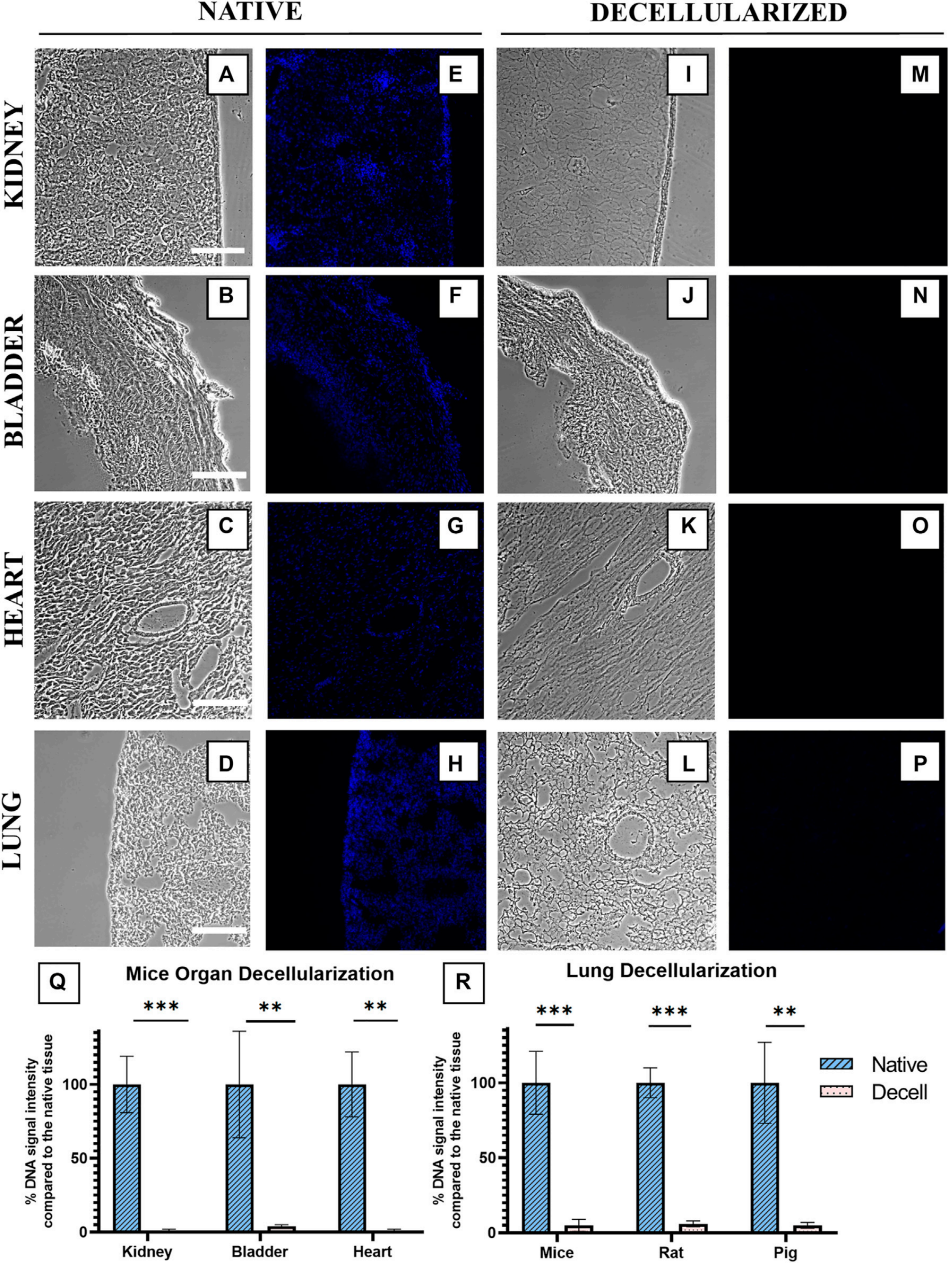
Effect of the decellularization treatment with SD on the DNA signal of tissues of different animal origins and organs. Phase contrast **(A–D,I–L)** and Hoechst 33342 nuclei stained images **(E–H,M–P)** of native **(A–H)** and decellularized **(I–P)** sections of kidney, bladder, heart, and lung. Quantification of different organ DNA content percentage (%) **(Q)** and different animal lung tissue DNA content percentage (%) **(R)** after decellularization treatment with SD. Native tissue’s DNA signal is normalized to 100%.

The quantification of the decellularization level of the mice’s bladder, heart, and kidney is displayed in Figure 5, showing that all organs were successfully decellularized. The decellularization level of the sections was quantified as described previously. Bladder decellularized sections had a mean value of 4% ± 1% of the native DNA signal intensity; kidney and heart decellularized sections both resulted in the same DNA intensity value of 1 ± 1% of the native DNA signal intensity value. It is important to note that, unlike lung samples, heart, kidney, and bladder samples remained attached for 100% of the decellularization trials. Porcine, rat, and mice lungs were decellularized and evaluated using the same protocol described previously. Lungs from the three different species were successfully decellularized (Figure 5). As expected, the decellularization of porcine and rat lung sections resulted in similar results to mice lung sections. Porcine lung sections averaged 5 ± 2% of the native DNA signal intensity while rat lung sections averaged 6 ± 2%. Mice lung decellularization with SD treatment was already described in Figure 1.

## 4 Discussion

In this work, we present an easy, reliable, and versatile method to decellularize tissue sections attached to a glass slide with the main advantage that it allows for the identification and comparison of biophysical and biochemical conditions before and after decellularization from small samples. A summary of the comparison between the different decellularization treatments can be found in Table 1. After comparing common strategies for tissue decellularization, SD resulted in the most efficient treatment to remove cells and maintain the ECM components. This method has been successfully tested for other tissues including the heart, bladder, and kidneys, and in different species such as porcine, murine (mice and rat) lung samples. The resulting scaffolds showed no mechanical differences to the corresponding native sections, and could be repopulated with MSCs with high viability rates for at least 72 h. Therefore, this novel decellularization method provides a reliable alternative to current decellularization protocols due to its simplicity, lower sample mass requirements, and most importantly, it opens up new research opportunities to understand the role of the ECM in some physiologic and pathological conditions as well as for tissue repair and engineering.

**TABLE 1:** Summary of results (%DNA retention, % sample attachment, %collagen, elastin and laminin retention) for the different decellularization methods.

Decellularization method	DNA retention, %	Sample attachment, %	Elastin retention, %	Collagen I retention, %	Laminin retention, %
Ammonium hydroxide 0.5% + Triton 0.1%	57.4	100.0	13.2	112.5	135.5
CHAPS 0.5%	61.8	82.1	39.7	110.6	125.8
Sodium Deoxycholate (SD) 2%	6.5	60.0	33.4	118.0	136.6
Sodium Dodecyl Sulfate (SDS) 1%	0.95	32.1	6.2	121.5	115.8
Triton X-100 1%	66.8	100.0	40.0	101.3	106.8
Trypsin 0.05% + EDTA 0.02%	—	0	—	—	—

Most of the available protocols for tissue decellularization are designed to decellularize whole organs or individual pieces of tissues not attached to a surface (da Palma et al., 2015; Urbano et al., 2017). Also, these protocols have been optimized for each organ and tissue type considering their structure and composition. To obtain a novel method capable to decellularize tissue samples attached to a glass slide, we compared and simplified the most common available protocols involving different techniques including perfusion, orbital agitation, and tissue immersion. The concentration employed for each decellularizing agent was the one previously optimized for lung tissues (see Section 2.2 and Supplementary Materials). From all decellularizing agents included in this study, only SD and SDS were able to remove efficiently the cells. Although SDS is the most effective treatment (1 ± 0.5% remaining native DNA), it was also very aggressive resulting in a marked ECM degradation, particularly in elastin. In contrast to SDS, treatment with SD preserved much better the ECM structure and composition maintaining a high level of cell removal (6.5 ± 5.8% of native DNA). In addition, collagen I and III staining using Picro-Sirius red indicated a reduction in these proteins in samples treated with SDS (81%) but not with other treatments. Since immunostaining of collagen type I did not show such decrease, this reduction suggests a removal of collagen type III specifically in SDS treatment. Furthermore, samples treated with SDS remained attached to the glass slide only 32 ± 22% compared to the 60 ± 23% of sample attachment after SD treatment. Thus, the selected decellularization process achieved an optimal balance between cell removal, ECM integrity and sample attachment.

The degradation/preservation of each ECM component depends on the decellularizing strategy employed. The more aggressive behavior of SDS shown here is in agreement with previous works. For instance, SDS promotes the denaturalization of the triple-helical collagen molecule while CHAPS and SD do not (Hwang et al., 2017) and reduce drastically the content of glycosaminoglycans (GAGs) (Ren et al., 2013). Additionally, White et al. found that SDS treatments lead to atypical phenotype, lower viability, and a reduced confluence of urothelial cells on decellularized scaffolds in contrast to SDS, Triton, and CHAPS treatments (White et al., 2017). Regarding ECM composition, none of the protocols tested in this work decreased the among of type I collagen or laminin. Elastin, which is known to be sensitive to the decellularization process (Maghsoudlou et al., 2013; O’Neill et al., 2013b; Alshaikh et al., 2020), was reduced for all decellularization methods tested. SDS and Ammonia + Triton, reduced the native elastin signal to 6%–13% while the other methods were able to preserve it better (30–40%). The sharp reduction in elastin signal was not hand in hand with levels of cellular removal; SDS was able to remove virtually all cellular material while the Ammonia + Triton mixture did not achieve sufficient decellularization levels (67 ± 28% of native DNA remained). This evidence suggests that the elastin decrease is influenced by the specific chemical interactions between decellularizing agents and the ECM network and not simply by the decellularization procedure. Additionally, by comparing the effect of the procedure on different organs, the elastin of bladder samples was highly preserved using the same SD based protocol, where 95 ± 36% of the native elastin signal was measured after decellularization (Supplementary Figure S3). Thus, elastin degradation seems to be organ-dependent and implicitly depend on the structure and organization of the ECM. Considering all these observations, although SDS is also extremely efficient in the removal of cellular material, we selected SD as the best option in terms of ECM preservation.

When comparing the effects of the decellularization procedure on the interstitial and basement membrane of the ECM, only a component of the interstitial ECM was significantly affected throughout procedures (elastin) while the component analyzed from the basement membrane (laminin) was not reduced in any of the treatments. This difference might be explained by the different locations of these layers: the interstitial ECM surrounds the cells and provides structural support to the tissue while the basement membrane is more compact (Cox and Erler 2011). This denser feature of the basement membrane might protect it from being removed during decellularization, while components from the interstitial ECM that are intertwined with the cells might be more vulnerable and disrupted when these are removed. Additionally, more components from both components of the ECM, like collagen type IV and fibronectin, could be analyzed for a more in-depth study on the effect of the decellularization procedure on the different layers of the ECM.

The key point in this novel method is to maintain the sample attached to the glass slide while applying the different decellularization solutions. Among all tissues employed, lungs have some unique features that could complicate their attachment. Specifically, lung scaffolds have large areas of empty spaces corresponding to the multiple alveoli and airways. These structures reduce the effective contact area between the scaffold and the glass slide in comparison to other more homogeneous tissues. While the lungs are being decellularized, the contact area with the glass slide can be further reduced by cellular removal leading to sample detachment during the different washes. For this reason, we selected lungs as the most difficult sample type to optimize the protocol. In fact, among all four organs tested in this work, the lungs were the only tissue where some samples would detach (40% for SD treatment) from the glass slide. Bladder, heart, and kidney samples remained attached to the glass slide for 100% of all experiments whilst reaching the same decellularization success using the same SD treatment. On the other hand, when testing solely lung samples, different detergents had dissimilar effects on the attachment of the slides. This could be explained by the effective contact area hypothesis just mentioned above, where the higher amount of cells removed, the smaller the contact area remaining with the glass slide and thus a higher chance of detachment. This is consistent with the results of this work, since the most effective decellularizing agents (SD and SDS) were less likely to preserve attachment when compared to less effective treatments, like triton and CHAPS. However, it is also worthy of note that ionic surfactants (SD and SDS) caused lower attachment levels when compared to non-ionic and zwitterionic surfactants (triton and CHAPS, respectively). Although the nature of this disruption is unclear, it is possible that the ionic nature of these reagents reacts with the ionic coating of the glass slides, disrupting it. We hypothesize that if this coating is replaced by a non-ionic treatment, attachment rates for treatments with SD and SDS might significantly improve.

Since this method maintains the sample attached to the glass slide, we were able to measure the mechanical properties of the same lung section before and after lung decellularization, unlike other protocols where mechanical properties are measured in different samples (Mendoza-Novelo et al., 2011; Maghsoudlou et al., 2013). In fact, it is possible to perform the decellularization *in situ* on the AFM stage, substantially reducing sources of variability. It is of the utmost importance that the scaffold maintains the mechanical properties of the native tissue, especially for cell culture and tissue engineering applications. As expected, tissue stiffness, viscosity and adhesion were maintained after decellularization in agreement with previous works that indicate that elastin does not play a significant role in the mechanics of the static lung (Guimarães et al., 2020).

One of the potential limitations of this method is the diffusion of the decellularizing agents through thicker samples as well as the increased chance of detachment, since with increased thickness the electrostatic bonds that secure the sample to the glass slide are not strong enough to retain the increased sample mass. To demonstrate the effectiveness in thicker sections, we tested the same protocol in 100 µm sections reaching similar decellularization levels by employing a longer incubation period of DNAse of 40 min at 37°C. It should be noted however, that incubation at higher temperatures may result in increased sample detachment (Eckhard et al., 2019). Thus, we would advise the use of additional adhesion treatments that form stronger bonds between the sample and the glass slide, such as transglutaminase or cellTAK. Both these compounds have been used in the past to secure tissues to surfaces for AFM measurements (Sahai et al., 2016). Notwithstanding the potential applications of this method to thicker tissue sections, one of the main advantages of the method described here is the fact that the decellularization of tissue slice by slice requires a minimal amount of tissue and effort. One of the main problems with the decellularization of organs from humans and large animals like the pig is the complex procedure to decellularize the whole organ that can weight more than 1 kg. This process usually requires organ-specific decellularizing reagents which also are applied by different approaches (vascular/airways perfusion, site injections, among others) (Balestrini et al., 2015; Pouliot et al., 2016). Here, we tested the decellularization method for lung slices with samples from mice, rats, and pigs obtaining similar results. Thus, this will open the opportunity to decellularize tissue slices from small clinical biopsies where the whole or a large section of an organ is not available.

In conclusion, the decellularization method presented here will provide a useful tool for many research aims, but mainly in 1) studies that require the identification of anatomical structures which can only be detected in native samples. For instance, the location of small metastasis in decellularized tissue samples may present a challenge since cancer cells have been removed. Since this novel approach provides access to the sample before and after decellularization in the same or consecutive slices, the native structure can be located before decellularization. The low mass requirements make this protocol an important tool for 2) studies of scarce and valuable samples, since sections as low as 10 µm could be decellularized with this protocol, unlike full organ or tissue submergence decellularization protocols. This is the case for clinical biopsies or smaller structures like the cornea. And 3) to use the decellularized ECM as a cell culture substrate. To this end, the biocompatibility of the scaffolds was also assessed after 72 h. The decellularized lung scaffolds showed the capability of supporting cell attachment and growth similar to the conventional culture conditions, as further evidenced by actin staining showing the spread morphology of the cells within the scaffold. In addition, we observed that cells seeded on this lung scaffold did not form a monolayer in the surface but reached different depths within the decellularized ECM opening new options for tissue repair and engineering.

## Data Availability

The raw data supporting the conclusion of this article will be made available by the authors, without undue reservation.
