# Supplementary material for: Novel Decellularization Method for Tissue Slices
Source: Front Bioeng Biotechnol. 2022 Mar 9;10:832178. doi: 10.3389/fbioe.2022.832178 (PMC8959585; doi:10.3389/fbioe.2022.832178)
Supplement: Supplementary file 1 [file Table1.DOCX]

Supplementary Material

# Supplementary Table 1

Decellularization protocols described in the literature used to establish the decellularization methods compared in this work.

| **Method** | **Decellularizing Agent (DA)** | **Sample** | **Delivery of DA** | **Duration** | **References** |
| --- | --- | --- | --- | --- | --- |
| **1** | Ammonium Hydroxide 0.5% + Triton 0.1% | Deposited ECM from cardiac cell culture | Static incubation of DA over cell culture petri dish | <1h | (Ng et al. 2019) |
| **2** | CHAPS 0.5% | 350µm lung tissue slices | Loose sections submerged in solution with agitation | 5-6h | (Rosmark et al. 2018) |
| **3** | Sodium Deoxycholate (SD) 2% | Whole lungs | Solutions injected through the trachea and incubated for hours. | 1-2 days | (Xiong et al. 2015) |
| **4** | Sodium Dodecyl Sulfate (SDS) 1% | Whole lungs | Perfusion of solutions through the trachea and vasculature | 5-6h | (Jorba et al. 2019) |
| **5** | Triton X-100 1% | Blocs of pericardium | Submersion of blocs in a decellularization solution under orbital agitation | 1-2 days | (Mendoza-Novelo et al. 2011) |
| **6** | Trypsin 0.05% + EDTA 0.02% | Heart valves | Full tissue submerged and DA incubated at constant agitation. | 1-3 days | (Schenke-Layland et al. 2003) |

# 
