# Supplementary material for: Novel Decellularization Method for Tissue Slices
Source: Front Bioeng Biotechnol. 2022 Mar 9;10:832178. doi: 10.3389/fbioe.2022.832178 (PMC8959585; doi:10.3389/fbioe.2022.832178)
Supplement: Supplementary file 2 [file Image2.pdf]

Supplementary Material

Supplementary Figures

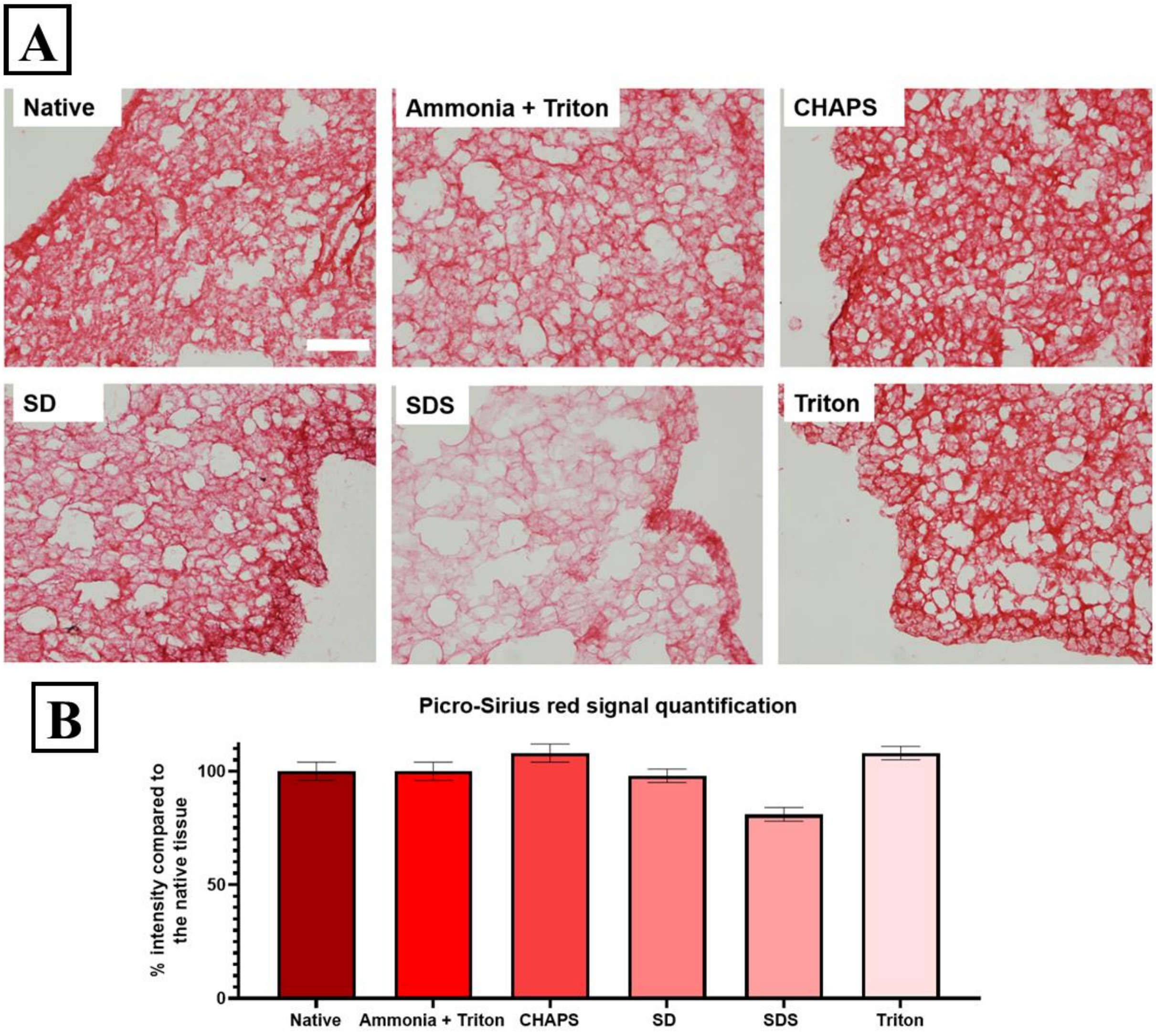

Supplementary Figure 2 – Picro-Sirius red staining for collagen I and III of 20µm mice lung sections treated with (A) Ammonia + Triton, CHAPS, SD, SDS and Triton or with no treatment (native). Scale bar = 100µm. (B) Signal quantification from picro-sirius red stained mice lungs from different decellularization treatments.
