# Supplementary material for: Novel Decellularization Method for Tissue Slices
Source: Front Bioeng Biotechnol. 2022 Mar 9;10:832178. doi: 10.3389/fbioe.2022.832178 (PMC8959585; doi:10.3389/fbioe.2022.832178)
Supplement: Supplementary file 3 [file Image3.pdf]

Supplementary Material

Supplementary Figures

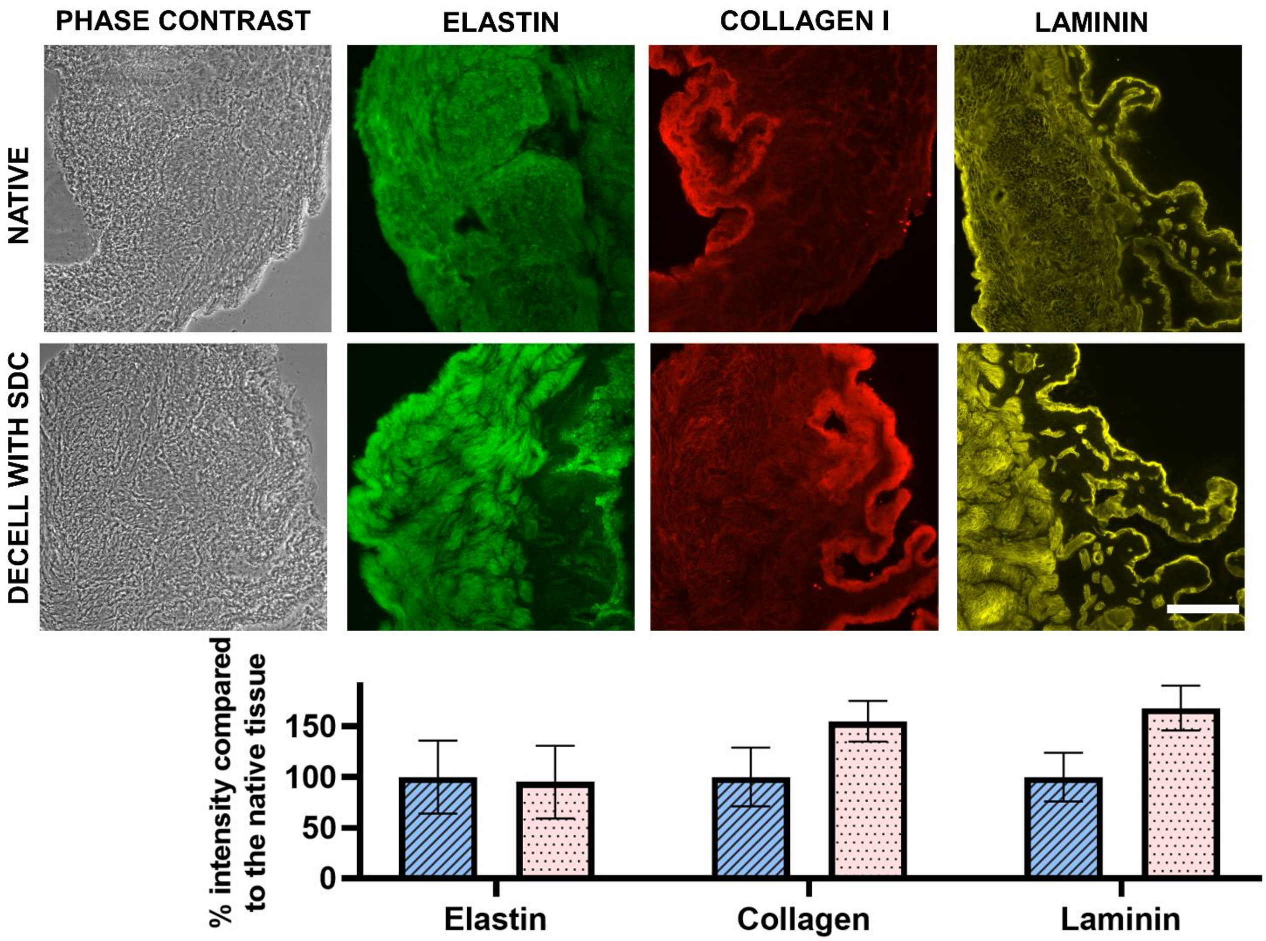

Supplementary Figure 3 - Staining and signal quantification of 20µm bladder sections from mice for elastin, type I collagen, and laminin. Laminin and elastin fluorescent images were changed from the original red color to yellow and green respectively for easier image readability. Scale bar = 200µm.
