# Supplementary material for: Novel Decellularization Method for Tissue Slices
Source: Front Bioeng Biotechnol. 2022 Mar 9;10:832178. doi: 10.3389/fbioe.2022.832178 (PMC8959585; doi:10.3389/fbioe.2022.832178)
Supplement: Supplementary file 4 [file Image1.pdf]

Supplementary Material

Supplementary Figures

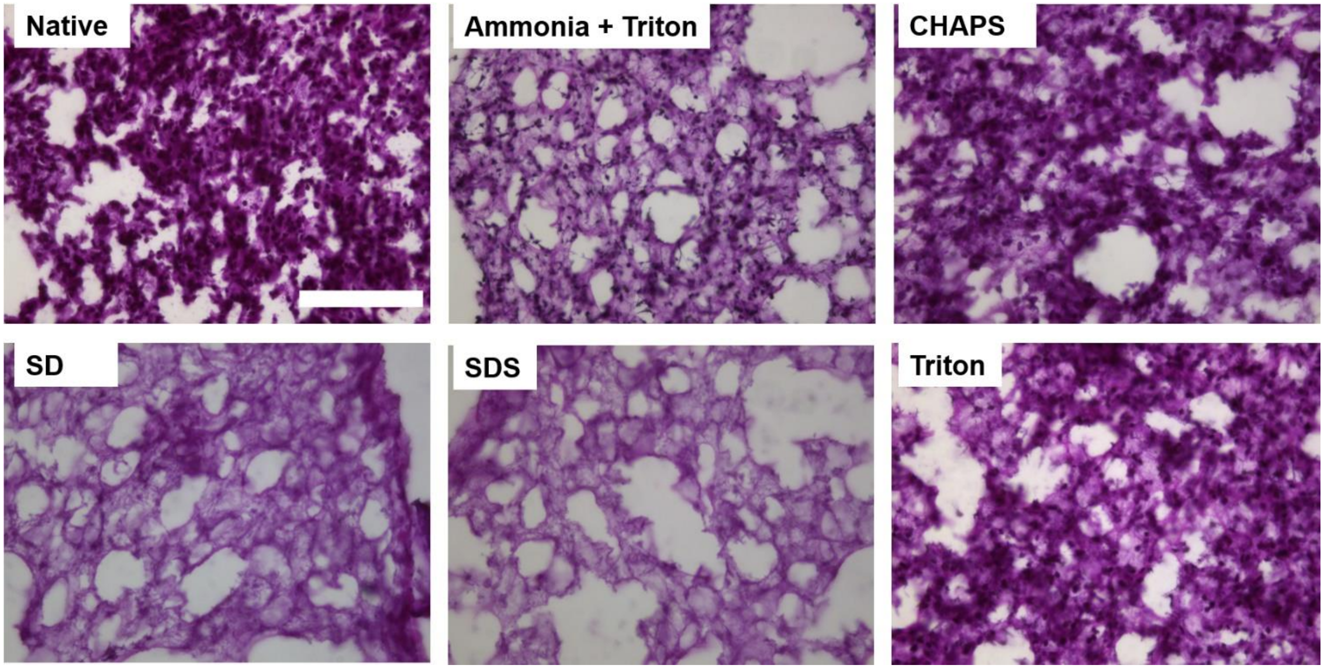

Supplementary Figure 1 – Hematoxylin and eosin (H&E) staining of 20µm mice lung sections treated with Ammonia + Triton, CHAPS, SD, SDS and Triton or with no treatment (native). Nuclei can be seen in dark purple. Scale bar = 100µm.
